# Correction: Dietary Protein Intake and Coronary Heart Disease in a Large Community Based Cohort: Results from the Atherosclerosis Risk in Communities (ARIC) Study

**DOI:** 10.1371/journal.pone.0114114

**Published:** 2014-11-20

**Authors:** The PLOS ONE Staff

## Notice of Republication

This article was republished on October 28, 2014, due to an error in the title. The publisher apologizes for this error. The citation has been updated to match the corrected title. Please download this article again to view the corrected title and citation. The originally published, uncorrected article and the republished, corrected article are provided here for reference.

## Supporting Information

File S1 **Originally published, uncorrected article.**
(PDF)

File S2 **Republished, corrected article.**
(PDF)
