# Supplementary material for: Correction: Dietary Protein Intake and Coronary Heart Disease in a Large Community Based Cohort: Results from the Atherosclerosis Risk in Communities (ARIC) Study
Source: PLoS One. 2014 Nov 20;9(11):e114114. doi: 10.1371/journal.pone.0114114 (PMC4239087; doi:10.1371/journal.pone.0114114)
Supplement: File S2 — Republished, corrected article. (PDF) [file pone.0114114.s002.pdf]

PLOS ONE

Dietary Protein Intake and Coronary Heart Disease in a Large Community Based Cohort: Results from the Atherosclerosis Risk in Communities (ARIC) Study

Bernhard Haring[1]*, Noelle Gronroos[2], Jennifer A. Nettleton[3], Moritz C. Wyler von Ballmoos[4], Elizabeth Selvin[5], Alvaro Alonso[2]

1 Department of Internal Medicine I, Comprehensive Heart Failure Center, University of Würzburg, Würzburg, Bavaria, Germany, **2** Division of Epidemiology and Community Health, University of Minnesota, Minneapolis, Minnesota, United States of America, **3** Division of Epidemiology, Human Genetics and Environmental Sciences, School of Public Health, University of Texas Health Science Center at Houston, Houston, Texas, United States of America, **4** Department of Surgery & Division of Cardiothoracic Surgery, Froedtert Memorial Hospital & Medical College of Wisconsin, Milwaukee, Wisconsin, United States of America, **5** Department of Epidemiology and the Welch Center for Prevention, Epidemiology and Clinical Research, Johns Hopkins Bloomberg School of Public Health, Baltimore, Maryland, United States of America

Abstract

Background: Prospective data examining the relationship between dietary protein intake and incident coronary heart disease (CHD) are inconclusive. Most evidence is derived from homogenous populations such as health professionals. Large community-based analyses in more diverse samples are lacking.

Methods: We studied the association of protein type and major dietary protein sources and risk for incident CHD in 12,066 middle-aged adults (aged 45–64 at baseline, 1987–1989) from four U.S. communities enrolled in the Atherosclerosis Risk in Communities (ARIC) Study who were free of diabetes mellitus and cardiovascular disease at baseline. Dietary protein intake was assessed at baseline and after 6 years of follow-up by food frequency questionnaire. Our primary outcome was adjudicated coronary heart disease events or deaths with following up through December 31, 2010. Cox proportional hazard models with multivariable adjustment were used for statistical analyses.

Results: During a median follow-up of 22 years, there were 1,147 CHD events. In multivariable analyses total, animal and vegetable protein were not associated with an increased risk for CHD before or after adjustment. In food group analyses of major dietary protein sources, protein intake from red and processed meat, dairy products, fish, nuts, eggs, and legumes were not significantly associated with CHD risk. The hazard ratios [with 95% confidence intervals] for risk of CHD across quintiles of protein from poultry were 1.00 [ref], 0.83 [0.70–0.99], 0.93 [0.75–1.15], 0.88 [0.73–1.06], 0.79 [0.64–0.98], P for trend = 0.16). Replacement analyses evaluating the association of substituting one source of dietary protein for another or of decreasing protein intake at the expense of carbohydrates or total fats did not show any statistically significant association with CHD risk.

Conclusion: Based on a large community cohort we found no overall relationship between protein type and major dietary protein sources and risk for CHD.

Citation: Haring B, Gronroos N, Nettleton JA, Wyler von Ballmoos MC, Selvin E, et al. (2014) Dietary Protein Intake and Coronary Heart Disease in a Large Community Based Cohort: Results from the Atherosclerosis Risk in Communities (ARIC) Study. PLoS ONE 9(10): e109552. doi:10.1371/journal.pone.0109552

Editor: Antony Bayer, Cardiff University, United Kingdom

Received May 7, 2014; **Accepted** September 4, 2014; **Published** October 10, 2014

Copyright: © 2014 Haring et al. This is an open-access article distributed under the terms of the Creative Commons Attribution License, which permits unrestricted use, distribution, and reproduction in any medium, provided the original author and source are credited.

Data Availability: The authors confirm that all data underlying the findings are fully available without restriction. All data underlying the findings in our study are freely available in a public repository governed by the 'Biologic Specimen and Data Repository Information Coordinating Center (BioLINCC)' under the auspices of the National Heart, Lung and Blood Institute (NHLBI). The repository can be accessed online (https://biolincc.nhlbi.nih.gov/studies/aric/?q = aric).

Funding: The Atherosclerosis Risk in Communities Study is carried out as a collaborative study supported by National Heart, Lung, and Blood Institute contracts (HHSN268201100005C, HHSN268201100006C, HHSN268201100007C, HHSN268201100008C, HHSN268201100009C, HHSN268201100010C, HHSN268201100011C, and HHSN268201100012C). There are no relationships with industry to declare. Open Access publication was funded by the German Research Foundation (DFG) and the University of Würzburg in the funding program Open Access Publishing. The funders had no role in study design, data collection and analysis, decision to publish, or preparation of the manuscript.

Competing Interests: The authors have declared that no competing interests exist.

* Email: Haring_B@ukw.de

Introduction

The relationship of dietary protein distinguished by animal versus vegetable origin with risk of coronary heart disease (CHD) has shown conflicting results [1,2,3,4,5,6]. This is surprising since the type of protein has been shown to influence cardiovascular risk factors such as hypertension [7,8,9,10,11,12]. Various observational studies [7,8,10,13] and feeding trials [9,11,12] have associated dietary protein of vegetable type inversely with blood pressure. To elucidate this apparent paradox, Bernstein et al. have focused on the effect of various food groups as major sources of dietary protein rather than on protein type in the Nurses' Health Study [2]. Greater consumption of red meat or processed meat

products was associated with a higher risk of CHD, while higher intakes of poultry, fish and nuts were associated with lower risk [2]. These findings are again in contrast with other results that showed no association of red meat with CHD [14] or that showed beneficial effects of animal protein on vascular health [15,16]. The discordance in findings may be explained by several factors including research design and study populations. Large randomized controlled feeding trials on this topic are sparse and current evidence is mostly derived from observational studies which used nurses or health professionals as study populations [1,2,4,5]. Community based analyses are mostly missing [6,17]. Thus, conclusions regarding the relation of various sources of protein intake with cardiovascular health are difficult to draw. Analyses conducted in large general communities are warranted as these may provide greater exposure variability with more generalizable results.

In this study, we aimed to investigate the associations between total, animal, and plant-based dietary protein, as well as individual protein-rich food groups, and the risk for CHD in a large, community-based cohort of middle-aged adults. We hypothesized that intake of animal protein and proteins from processed meats would be associated with a higher risk of CHD and vegetable proteins and corresponding food groups with a lower CHD risk.

Methods

Study Population

The Atherosclerosis Risk in Communities Study (ARIC) is a community-based prospective cohort study of 15,792 middle-aged adults (aged 45–64 years at baseline) from four U.S. communities (Washington County, Md; Forsyth County, NC; Jackson, Miss; and suburbs of Minneapolis, Minn.) [18]. The first examination (visit 1) of participants occurred during 1987–1989, with three follow-up visits taking place each approximately every 3 years; response rates were 93%, 86%, and 81% at visits 2 (1990 to 1992), 3 (1993 to 1995), and 4 (1996 to 1998), respectively. A fifth exam (visit 5) took place in 2011–2013 among surviving participants. At all visits, participants received an extensive examination, including collection of medical, social, and demographic data [18]. For this analysis, only white and black adults were included; blacks from the Minneapolis and Washington County field centers were excluded due to small numbers. Individuals with self-reported diabetes, fasting blood glucose ≥126 mg/dL, non-fasting blood glucose ≥200 mg/dL or use of diabetes medication, a history of myocardial infarction, stroke, heart failure, coronary bypass surgery, angioplasty or with missing data on covariates of interest were excluded. Our final sample size included 12,066 persons.

The ARIC study was approved by the Institutional Review Boards (IRB) of all participating institutions, including the IRBs of the University of Minnesota, Johns Hopkins University, University of North Carolina, University of Mississippi Medical Center, and Wake Forest University. Written informed consent at each clinical site was obtained from all participants.

Assessment of protein intake

Protein intake was assessed using an interviewer-administered, 66-item food frequency questionnaire (FFQ) adapted from the 61-item FFQ developed by Willett et al. [19]. The FFQ was administered to all subjects at visit 1 at baseline (1987–1989) and at visit 3 (1993–1995). The usual frequency of food consumption was reported in 9 categories, from never or less than once a month to>6 times per day. The major contributors to protein intake included: unprocessed red meat, processed red meat, poultry, high-fat dairy, low-fat dairy, fish & seafood, eggs, nuts, and legumes. Average daily intake of nutrients was calculated by multiplying the frequency of consumption of each food item by its nutrient content and adding up the nutrient intake for all of the items. Vegetable protein intake was defined as the difference of total and animal protein intake. The residual method was used to adjust for total energy intake [20]. For assessing dietary behaviour, participants were divided into quintiles of cumulative average intake of various protein sources. Cumulative updating of the FFQ (i.e. visit 1 FFQ for follow-up between visit 1 and visit 3 and the average of visits 1 and 3 FFQ afterwards for those who attended both examinations, or visit 1 FFQ for those who did not attend visit 3) was used to reduce within-person variation and best represent long-term dietary behavior [2]. Participants with incomplete dietary information or with extreme calorie intake (<600 kcal or>4200 kcal per day for men, <500 kcal or> 3600 kcal per day for women) were excluded from further analysis. We stopped updating a participant's cumulative average intake when the participant of our study was diagnosed with an intermediate variable on the causal pathway between diet and CHD such as hypercholesterolemia, hypertension, stroke and diabetes. This was done to avoid exposure misclassification due to short-term changes in dietary patterns.

Assessment of coronary heart disease

The primary end point for this study was CHD occurring after the completion of the first FFQ (between 1987 and 1989). CHD was defined as a definite or probable myocardial infarction or a death from coronary heart disease. CHD events were identified and adjudicated using information from study visits, yearly telephone follow-up calls, review of hospital discharge lists and medical charts, death certificates, next-of-kin interviews, and physician-completed questionnaires [18,21]. Follow-up for CHD was available until December 31, 2010.

Covariates

Height, weight, and waist circumference were measured following a standardized protocol [18,21]. ARIC participants underwent fasting venipuncture at each examination [18]. Diabetes was defined as current use of glucose-lowering medications, fasting blood glucose ≥126 mg/dL, non-fasting blood glucose ≥200 mg/dL or self-reported history of diabetes. Hypertension was defined as the average of the last two of three blood-pressure readings at the first visit (using 140 mmHg or higher for systolic and 90 mmHg or higher for diastolic as cut-off points). Current smoking, ethanol intake, education, intake of antihypertensive or lipid lowering medication were derived from standardized questionnaires [18]. Sports-related physical activity and leisure related physical activity were assessed with the use of Baecke's questionnaire and scoring systems [22].

Statistical Analysis

To assess the association of CHD and average cumulative intake of protein by quintiles, we calculated incidence rates (IR) of CHD events per 1000 person-years as the number of diagnosed cases of CHD occurring during the entire follow-up period divided by person-years of follow-up. Person-years of follow up were defined as time from the baseline examination to the date of the first coronary event, death, lost to follow-up, or December 31, 2010, whichever occurred earlier. Thereafter, corresponding rate ratios were calculated by dividing the rate among participants in each specific intake quintile by the rate among participants in the lowest quintile of intake (reference). Cox proportional hazards regression models were used to account for potential confounding. An initial model adjusted for age, race, sex, ARIC study center,

Table 1. Unadjusted baseline characteristics according to quintiles of total protein intake, ARIC 1987–1989.

	Q 1 (low)	Q 2	Q 3	Q 4	Q5 (high)	p-trend[a]
N	2412	2414	2413	2414	2413	
Protein intake, g/day (SD)	49.3 (10.2)	62.9 (3.9)	70.2(3.9)	77.8 (4.0)	93.5 (12.6)	<0.0001
Protein intake, % of total energy	12.4 (1.7)	15.7 (1.0)	17.8 (1.5)	19.8 (2.0)	22.8 (3.4)	<0.0001
Age, years (SD)	53.4 (5.7)	54.0 (5.7)	54.0 (5.8)	53.8 (5.7)	53.8 (5.7)	0.11
Women, %	55.9	55.8	55.8	55.8	55.8	0.99
Black, %	24.8	24.5	21.9	21.6	22.4	0.005
High school graduate, %	72.1	77.7	80.7	82.6	84.0	<0.0001
Current smoker, %	32.3	28.8	24.7	22.5	21.6	<0.0001
Hypertension, %	29.9	27.5	29.1	28.0	28.8	0.57
Body Mass Index, kg/m^2 (SD)	26.6 (5.1)	26.8 (4.9)	27.0 (5.0)	27.4 (5.0)	27.7 (5.1)	<0.0001
Waist-to-hip ratio (SD)	0.9 (0.1)	0.9 (0.1)	0.9 (0.1)	0.9 (0.1)	0.9 (0.1)	0.19
Baecke Sport Activity Score (SD)	2.4 (0.8)	2.4 (0.8)	2.5 (0.8)	2.5 (0.8)	2.6 (0.8)	<0.0001
Baecke Leisure Index (SD)	2.3 (0.6)	2.3 (0.6)	2.4 (0.6)	2.4 (0.6)	2.5 (0.6)	<0.0001
Systolic blood pressure, mmHg (SD)	120.7 (18.7)	119.9 (17.5)	119.9 (17.6)	119.2 (17.6)	119.4 (18.2)	0.004
Serum HDL, mmol/L (SD)	1.4 (0.4)	1.4 (0.4)	1.4 (0.4)	1.4 (0.5)	1.4 (0.4)	0.27
Serum cholesterol, mmol/L (SD)	5.5 (1.0)	5.5 (1.1)	5.6 (1.1)	5.5 (1.1)	5.5 (1.1)	0.23
Use of antihypertensive medication, %	19.4	18.3	20.4	18.4	19.6	0.89
Use of lipid lowering medication, %	1.3	1.7	2.2	3.2	2.7	<0.0001
Carbohydrate intake, g/day (SD)	231.2 (57.6)	208.0 (31.4)	197.8 (31.3)	188.5 (32.3)	172.4 (38.4)	<0.0001
Carbohydrate intake, % of total energy (SD)	56.2 (9.5)	51.3 (7.9)	48.3 (7.7)	45.8 (7.5)	42.8 (7.8)	<0.0001
Fiber intake, g/day (SD)	15.5 (7.4)	16.7 (5.6)	17.3 (5.6)	17.6 (5.9)	18.3 (7.0)	<0.0001
Magnesium intake, g/day (SD)	217.0 (66.1)	240.7 (52.5)	253.5 (53.9)	267.0 (57.9)	288.4 (65.6)	<0.0001
Alcohol intake, g/week (SD)	68.7 (148.7)	43.9 (89.0)	40.7 (78.9)	36.9 (70.4)	32.7 (65.4)	<0.0001
Total energy intake, kcal/day (SD)	1818.1 (693.9)	1488.7 (555.6)	1489.7 (538.1)	1565.9 (537.1)	1802.8 (606.7)	0.23
Total fat Intake (g/d) (Median ±SD)	53.9 (16.0)	59.9 (11.7)	61.4 (12.0)	62.6 (12.8)	63.4 (15.4)	<0.0001
Total fat intake, % of total energy (SD)	30.0 (7.2)	32.5 (6.2)	33.4 (6.3)	34.2 (6.2)	34.5 (6.5)	<0.0001

Values are % for categorical variables and mean (SD) for continuous variables.
[a]p-values from general linear models for continuous variables and Mantel-Haenszel 1-degree of freedom chi-square statistic.
doi:10.1371/journal.pone.0109552.t001

and total energy intake (minimally adjusted model). A second model additionally adjusted for smoking (current, former, never), pack years of smoking, education (less than high school, high school, more than high school), systolic blood pressure (mmHg), use of antihypertensive medication, HDLc (mmol/l), total cholesterol (mmol/l), use of lipid lowering medication, body mass index (kg/m^2), waist-to-hip ratio, alcohol intake (g/week), Baecke's physical activity score, leisure-related physical activity, carbohydrate intake (quintiles), fiber intake (quintiles), and magnesium intake (quintiles) (fully adjusted model). Median protein intake of each quintile (g/d) modeled as a continuous variable was used to test for linear trend.

We further conducted food substitution analyses based on the fully adjusted model. Hazard ratios of CHD associated with increasing 1 serving/day in the consumption of protein sources at the expense of decreasing 1 serving/day in a different protein source were calculated. Similarly, we conducted nutrient substitution analyses by examining the risk for CHD when increasing 10% energy from carbohydrates or fat while decreasing 10% of energy from protein. Tests of the proportional hazards assumption were evaluated. All p-values were 2-tailed. Data were analyzed with SAS 9.3 (SAS Corp, Cary, NC).

Results

Baseline characteristics of the study participants according to quintiles of total protein intake at baseline are shown in Table 1. Compared with participants with low protein consumption, individuals with high protein consumption were less likely to be current smoker, to drink less alcohol per week, and more likely to conduct physical activity and to have graduated from high school. Furthermore, participants with high protein intake had higher BMI levels, higher intakes of fiber, magnesium and fat whereas decreased intake of carbohydrates.

During a median follow-up of 22 years, there were 1,147 CAD events among the 12,066 participants at baseline. In age, sex, race, study center and total energy adjusted analyses (minimally adjusted model) animal protein intake was not associated with an increased risk for CHD (Table 2). These results did not change significantly after full adjustment. In the minimally adjusted model, total and vegetable protein were associated with a significantly lower risk of CHD (Table 2, Model 1). This relationship was considerably attenuated and became non-significant after full adjustment (Table 2, Model 2).

In food-group analyses of major dietary protein sources using our minimally adjusted model, higher intake of red or processed meat was significantly associated with increased risk for CHD,

Table 2. Association of total, animal and vegetal protein intake with coronary heart disease incidence, ARIC 1987–2010.

	Q 1	Q 2	Q 3	Q 4	Q 5	p-trend
**Total Protein Intake**						
Events, n	241	230	231	230	215	
Person-time	46149	46720	46725	46991	47102	
Incidence, per 1000 py	5.2	4.9	4.9	4.9	4.6	
HR (95%CI)*	1 (ref)	0.84 (0.70, 1.01)	0.86 (0.72, 1.03)	0.89 (0.74, 1.06)	0.79 (0.66, 0.95)	0.04
HR (95%CI)**	1 (ref)	0.91 (0.75, 1.11)	0.93 (0.76, 1.14)	1.00 (0.80, 1.24)	0.84 (0.66, 1.07)	0.34
**Animal Protein Intake**						
Events, n	236	240	212	238	221	
Person-time	46175	46781	46915	47067	46750	
Incidence, per 1000 py	5.1	5.1	4.5	5.1	4.7	
HR (95%CI)*	1 (ref)	1.05 (0.88, 1.26)	0.97 (0.80, 1.17)	1.01 (0.84, 1.21)	0.95 (0.79, 1.15)	0.56
HR (95%CI)**	1 (ref)	1.16 (0.96, 1.40)	1.01 (0.82, 1.24)	1.11 (0.90, 1.37)	1.00 (0.79, 1.26)	0.94
**Vegetable Protein Intake**						
Events, n	247	228	253	215	204	
Person-time	45991	46902	46518	47070	47207	
Incidence, per 1000 py	5.4	4.9	5.4	4.6	4.3	
HR (95%CI)*	1 (ref)	0.92 (0.77, 1.10)	0.91 (0.76, 1.09)	0.82 (0.68, 0.98)	0.71 (0.59, 0.85)	0.0001
HR (95%CI)**	1 (ref)	1.08 (0.89, 1.31)	1.15 (0.94, 1.40)	1.04 (0.84, 1.29)	0.87 (0.68, 1.10)	0.17

*adjusted for age, sex, race, study, center, and total energy intake.
** adjusted for age, sex, race, study center, total energy intake, smoking, education, systolic blood pressure, use of antihypertensive medication, HDLc, total cholesterol, use of lipid lowering medication, body mass index, waist-to-hip ratio, alcohol intake, sports-related physical activity, leisure-related physical activity, carbohydrate intake, fiber intake, and magnesium intake.
doi:10.1371/journal.pone.0109552.t002

whereas low-fat dairy, poultry and nuts consumption were significantly associated with decreased risk for CHD (Table 3, Model 1). After adjustment for potential confounders, only higher poultry intake remained associated with a lower risk of CHD (Table 3, Model 2).

Last, we conducted replacement analysis evaluating the association of substituting one source of dietary protein for another and of decreasing protein intake at the expense of carbohydrates or total fats (Table 4). Overall, these results did not show any statistically significant association with CHD risk, although they suggested that decreasing red meat and increasing intake of any other protein source was associated with non-significant lower risk of CHD (10–20% reduction per 1 serving/day change).

Discussion

In this prospective community based study with 22 years of follow-up, neither total, animal or vegetable protein intake was associated with risk of CHD. In food group analyses of major dietary protein sources, we found no significant trend between various sizes of intake of meat products, poultry, dairy, eggs, nuts, fish, or legumes and risk for CHD. Our results are contrary to our initial hypothesis as we expected food groups based on animal protein such as red or processed meat products to be significantly associated with an increased risk for CHD.

Thus far, the largest cohort analyses to examine an association between protein intake and coronary heart disease were undertaken using data from the Nurses' Health Study with 14, 16 and 26 years of follow up [2,5,23]. Interestingly, similar to our results it was found that after 14 to 16 years of follow-up, neither animal protein nor vegetable protein were associated with CHD [5,24]. Results from the Health Professional Follow-up Study also indicate no association between dietary protein and risk of coronary heart disease after 18 years of follow-up [4]. In a later analysis of the Nurses' Health Study spanning 26 years of follow-up higher intakes of red meat, red meat excluding processed meat, and high-fat dairy were indeed found to be significantly associated with an elevated risk of CHD while higher intakes of poultry, fish, and nuts were significantly associated with lower risk [2]. Other prospective studies using California Seventh Day Adventists or the NIH-AARP Diet and Health cohort as study base also report a positive association between (red) meat consumption and CHD risk [6,25]. Nonetheless, generalizability of the existing data is limited as the respective cohorts are characterized by well-educated, ethnically homogenous study populations. A recent meta-analysis summarizing 9 studies on red and processed meat consumption and risk for CHD found processed meats (RR 1.42, 95%CI 1.07, 1.89), but not red meats (RR 1.00, 95% CI 0.81,1.23) to increase incident coronary events [14]. The effects of other dietary protein sources or type of protein were not addressed in this analysis. Moreover all included studies were observational and among included studies only one was based in a general community setting in the UK [17]. Interventional studies such as the Bold Study suggest that dietary protein, also of animal origin, can exert positive effects on biomarkers of CHD [15,16]. Lean beef in an optimal lean diet has been shown to exhibit beneficial effects on systolic blood pressure and vascular elasticity [15,16].

In spite of the lack of strong epidemiologic evidence for an association between animal derived protein sources (in particular meat products) and risk for CHD, several arguments mainly based on contents of sodium and saturated fat have been previously made to potentially explain a harmful effect of animal derived protein products on the risk of CHD. Processed meats are known

Table 3. Association of major dietary protein sources with coronary heart disease, ARIC 1987–2010.

	Q 1	Q 2	Q 3	Q 4	Q 5	p-trend
**Processed Meat**						
Median svg/day	0	0.1	0.4	0.5	1.1	
HR (95%CI)*	1 (ref)	1.04 (0.86, 1.25)	1.22 (1.01, 1.49)	1.28 (1.06, 1.553)	1.40 (1.15, 1.71)	0.003
HR (95%CI)**	1 (ref)	0.95 (0.78. 1.15)	1.02 (0.84, 1.24)	1.04 (0.85, 1.265)	1.04 (0.85, 1.29)	0.49
**Red Meat**						
Median svg/day	0.1	0.3	0.5	0.6	1.1	
HR (95%CI)*	1 (ref)	1.00 (0.83, 1.21)	1.11 (0.93, 1.33)	1.18 (0.98, 1.43)	1.30 (1.06, 1.59)	0.004
HR (95%CI)**	1 (ref)	0.92 (0.76, 1.12)	1.02 (0.85, 1.24)	1.10 (0.90, 1.35)	1.13 (0.89, 1.44)	0.13
**Red Meat & Processed Meat**						
Median svg/day	0.2	0.5	0.8	1.2	1.9	
HR (95%CI)*	1 (ref)	1.15 (0.95, 1.40)	1.32 (1.09, 1.60)	1.51 (1.24, 1.84)	1.53 (1.23, 1.90)	$<$0.0001
HR (95%CI)**	1 (ref)	1.00 (0.82, 1.23)	1.084 (0.88, 1.33)	1.18 (0.95, 1.46)	1.15 (0.89, 1.48)	0.21
**Poultry**						
Median svg/day	0.1	0.1	0.3	0.4	0.8	
HR (95%CI)*	1 (ref)	0.80 (0.67, 0.95)	0.83 (0.67, 1.03)	0.75(0.63, 0.90)	0.67 (0.55, 0.82)	0.0007
HR (95%CI)**	1 (ref)	0.83 (0.70, 0.99)	0.93 (0.75, 1.15)	0.88 (0.73, 1.06)	0.79 (0.64, 0.98)	0.16
**Dairy**						
Median svg/day	0.1	0.6	1.1	1.5	2.9	
HR (95%CI)*	1 (ref)	0.88 (0.73, 1.05)	0.99 (0.83, 1.18)	0.72 (0.59, 0.87)	0.88 (0.72, 1.08)	0.24
HR (95%CI)**	1 (ref)	0.96 (0.80, 1.16)	1.14 (0.95, 1.37)	0.85 (0.69, 1.04)	1.04 (0.84, 1.29)	0.77
**High-Fat Dairy**						
Median svg/day	0.1	0.1	0.4	0.8	1.2	
HR (95%CI)*	1 (ref)	1.09 (0.91, 1.31)	0.96 (0.80, 1.16)	1.04 (0.86, 1.27)	1.1 (0.90, 1.34)	0.56
HR (95%CI)**	1 (ref)	1.16 (0.96, 1.39)	1.03 (0.86, 1.25)	1.13 (0.93, 1.38)	1.14 (0.93, 1.39)	0.47
**Low-Fat Dairy**						
Median svg/day	0	0.1	0.4	1	2.5	
HR (95%CI)*	1 (ref)	0.93 (0.77, 1.12)	0.73 (0.61, 0.87)	0.73 (0.61, 0.87)	0.75 (0.62, 0.90)	0.007
HR (95%CI)**	1 (ref)	1.04 (0.86, 1.25)	0.86 (0.72, 1.03)	0.90 (0.75, 1.08)	0.91 (0.74, 1.12)	0.39
**Fish & seafood**						
Median svg/day	0	0.1	0.2	0.3	0.6	
HR (95%CI)*	1 (ref)	0.98 (0.81, 1.17)	1.05 (0.85, 1.29)	0.95 (0.77, 1.16)	0.90 (0.74, 1.10)	0.20
HR (95%CI)**	1 (ref)	1.04 (0.87, 1.25)	1.17 (0.95, 1.44)	1.07 (0.87, 1.32)	1.06 (0.86, 1.31)	0.81
**Eggs**						
Median svg/day	0	0.1	0.1	0.4	1.0	
HR (95%CI)*	1 (ref)	0.90 (0.74, 1.09)	0.86 (0.72, 1.03)	0.84 (0.70, 1.01)	1.09 (0.88, 1.34)	0.20
HR (95%CI)**	1 (ref)	0.92 (0.76, 1.12)	0.88 (0.73, 1.06)	0.83 (0.69, 0.99)	0.96 (0.77, 1.19)	0.89
**Nuts**						
Median svg/day	0	0.1	0.2	0.4	1.0	
HR (95%CI)*	1 (ref)	0.83 (0.70, 0.99)	0.74 (0.60, 0.90)	0.71 (0.59, 0.87)	0.73 (0.60, 0.89)	0.02
HR (95%CI)**	1 (ref)	0.89 (0.75, 1.06)	0.86 (0.71, 1.05)	0.83 (0.68, 1.01)	0.91 (0.74, 1.12)	0.67
**Legumes**						
Median svg/day	0.1	0.1	0.2	0.3	0.6	
HR (95%CI)*	1 (ref)	1.01 (0.84, 1.20)	1.04 (0.83, 1.32)	0.98 (0.82, 1.17)	1.04 (0.85, 1.26)	0.64
HR (95%CI)**	1 (ref)	1.07 (0.89, 1.27)	1.16 (0.92, 1.46)	1.05 (0.87, 1.27)	1.159 (0.93, 1.44)	0.18

*adjusted for age, sex, race, study, center, and total energy intake.
** adjusted for age, sex, race, study center, total energy intake, smoking, education, systolic blood pressure, use of antihypertensive medication, HDLc, total cholesterol, use of lipid lowering medication, body mass index, waist-to-hip ratio, alcohol intake, sports-related physical activity, leisure-related physical activity, carbohydrate intake, fiber intake, and magnesium intake.
doi:10.1371/journal.pone.0109552.t003

to have high sodium contents. High sodium intake is strongly correlated with the development of hypertension and CHD

Table 4. Food substitution analysis.

Increase 1 svg/d → Decrease 1 svg/d ↓	Red meat	Poultry	High-fat dairy	Low-fat dairy	Fish/seafood	Eggs	Nuts	Legumes
Processed meat	1.14 (0.90–1.44)	0.93 (0.73–1.18)	1.04 (0.90–1.20)	0.96 (0.84–1.11)	0.98 (0.74–1.15)	0.92 (0.74–1.15)	0.94 (0.79–1.10)	0.97 (0.74–1.27)
Red meat	-	0.81 (0.62–1.07)	0.91 (0.74–1.12)	0.84 (0.68–1.05)	0.86 (0.65–1.13)	0.81 (0.63–1.05)	0.81 (0.65–1.02)	0.85 (0.61–1.18)
Poultry	-	-	1.12 (0.90–1.40)	1.04 (0.83–1.30)	1.05 (0.76–1.45)	0.99 (0.77–1.29)	1.00 (0.79–1.27)	1.04 (0.76–1.44)
High-fat dairy	-	-	-	0.93 (0.84–1.02)	0.94 (0.75–1.19)	0.89 (0.75–1.06)	0.90 (0.78–1.03)	0.93 (0.73–1.19)
Low-fat dairy	-	-	-	-	1.02 (0.80–1.28)	0.96 (0.81–1.14)	0.97 (0.84–1.11)	1.01 (0.79–1.28)
Fish/seafood	-	-	-	-	-	0.94 (0.72–1.24)	0.95 (0.74–1.22)	0.99 (0.72–1.38)
Eggs	-	-	-	-	-	-	1.01 (0.83–1.22)	1.05 (0.79–1.39)
Nuts	-	-	-	-	-	-	-	1.04 (0.81–1.34)

Nutrient substitution analysis:
- Increasing 10% energy from carbohydrates, decreasing 10% from protein: HR 0.96 (95% CI 0.82–1.14).
- Increasing 10% energy from fats, decreasing 10% from protein: HR 0.99 (95% CI 0.80–1.24).

*adjusted for age, sex, race, study center, total energy intake, smoking, education, systolic blood pressure, use of antihypertensive medication, HDLc, total cholesterol, use of lipid lowering medication, body mass index, waist-to-hip ratio, alcohol intake, sports-related physical activity, leisure-related physical activity, carbohydrate intake, fiber intake, magnesium intake, and all the food items in the table (continuous variables, in servings/day).
Hazard ratios (95% confidence intervals) of CHD associated with increasing 1 serving/day in the consumption of protein sources (rows) at the expense of decreasing 1 serving/day in a different protein source (columns).* For example, the orange shaded cell can be interpreted as the HR (95% CI) of CHD increasing poultry consumption 1 serving/day and reducing red meat by the same amount, keeping all other sources of dietary protein constant.
doi:10.1371/journal.pone.0109552.t004

[26,27]. On the other hand, high animal protein as provided by dark meat intake often accompanies large intakes of saturated fat intake which has been linked to increased cardiovascular risk [23,28,29]. In contrast to animal protein diets consisting of vegetable protein have been associated with cardiovascular and overall health benefits because of their high content of mono- and polyunsaturated fats, fiber, vitamins and minerals and low content of sodium [30]. These lines of argumentation are supported by current dietary recommendations [31], however, the role of saturated fat on the risk of CHD has been subject to controversial debates most recently [32,33]. Further research is warranted to elucidate the mechanisms of action of protein on the risk of CHD.

The absence of an association between major dietary protein sources and risk for CHD in our population may be explained in part by limited variation in consumptions of these food groups. Our study participants reported low meat intake whereas consumption of eggs, nuts, fish and dairy consumption were similar to other study populations [2]. Therefore, our observation suggesting a protective association of high poultry intake with lower CHD risk has to be interpreted with caution as this singular result may be spurious.

Other reasons that explain inconsistent findings and reports between dietary protein sources, food groups and CHD can be found in differences in study design, follow-up and assessment of outcomes and covariates. Similar to previous reports of the Nurses' Health Study and the Health Professional Study a follow-up period of 22 years may not have been long enough to detect significant differences between various dietary intake levels and risk for CHD events [2,4,5]. Second, our dietary data assessment was imperfect and incomplete. Although repeated dietary data measurements may take changing dietary patterns into account and thus serve to reduce intra-individual error, exposure variability of our study population was limited as protein intake was only assessed at two time-points. Changing dietary habits may not have been covered adequately by our FFQs with time-points only 6 years apart. On the other hand, it is well known that behavioral dietary changes are very challenging to accomplish and to maintain on the individual level and thus great changes in the overall population are unlikely to occur [34,35]. Last, dietary substitution effects as well as different characteristics of particular food group (e.g fat content, micronutrient content) may limit our analyses. Strengths of our study include the sample size, a large community based cohort with two different races in the setting of the general US population and a prospective design with long follow-up. CHD and several confounding factors were assessed using standardized protocols whereas other studies were based on self-report data [2,5].

In conclusion, using a large community based cohort study we found neither total nor animal or vegetable protein to be associated with CHD. In detailed food group analyses of major protein sources, no statistically significant trends between animal or vegetable-based food groups and risk for CHD were observed. Individuals should continue to make appropriate dietary modifications following current guidelines and recommendations for cardiovascular disease risk reduction [16,36].

Acknowledgments

The authors thank the staff and participants of the ARIC study for their important contributions. AA, BH and NG had full access to all of the data in the study and take responsibility for the integrity of the data and the accuracy of the data analysis.

Author Contributions

Conceived and designed the experiments: BH NG JN MWvB ES AA. Performed the experiments: ES JN BH AA. Analyzed the data: AA NG BH. Contributed reagents/materials/analysis tools: JN NG ES BH MWvB AA. Wrote the paper: BH NG JN MWvB ES AA.

References

1. Halton TL, Willett WC, Liu S, Manson JE, Albert CM, et al. (2006) Low-carbohydrate-diet score and the risk of coronary heart disease in women. N Engl J Med 355: 1991–2002.
2. Bernstein AM, Sun Q, Hu FB, Stampfer MJ, Manson JE, et al. (2010) Major dietary protein sources and risk of coronary heart disease in women. Circulation 122: 876–883.
3. Kelemen LE, Kushi LH, Jacobs DR Jr, Cerhan JR (2005) Associations of dietary protein with disease and mortality in a prospective study of postmenopausal women. Am J Epidemiol 161: 239–249.
4. Preis SR, Stampfer MJ, Spiegelman D, Willett WC, Rimm EB (2010) Dietary protein and risk of ischemic heart disease in middle-aged men. Am J Clin Nutr 92: 1265–1272.
5. Hu FB, Stampfer MJ, Manson JE, Rimm E, Colditz GA, et al. (1999) Dietary protein and risk of ischemic heart disease in women. Am J Clin Nutr 70: 221–227.
6. Sinha R, Cross AJ, Graubard BI, Leitzmann MF, Schatzkin A (2009) Meat intake and mortality: a prospective study of over half a million people. Arch Intern Med 169: 562–571.
7. Stamler J, Elliott P, Kesteloot H, Nichols R, Claeys G, et al. (1996) Inverse relation of dietary protein markers with blood pressure. Findings for 10,020 men and women in the INTERSALT Study. INTERSALT Cooperative Research Group. INTERnational study of SALT and blood pressure. Circulation 94: 1629–1634.
8. Iseki K, Iseki C, Itoh K, Sanefuji M, Uezono K, et al. (2003) Estimated protein intake and blood pressure in a screened cohort in Okinawa, Japan. Hypertens Res 26: 289–294.
9. Stamler J, Caggiula A, Grandits GA, Kjelsberg M, Cutler JA (1996) Relationship to blood pressure of combinations of dietary macronutrients. Findings of the Multiple Risk Factor Intervention Trial (MRFIT). Circulation 94: 2417–2423.
10. Stamler J, Liu K, Ruth KJ, Pryer J, Greenland P (2002) Eight-year blood pressure change in middle-aged men: relationship to multiple nutrients. Hypertension 39: 1000–1006.
11. Wang YF, Yancy WS Jr, Yu D, Champagne C, Appel LJ, et al. (2008) The relationship between dietary protein intake and blood pressure: results from the PREMIER study. J Hum Hypertens 22: 745–754.
12. Appel LJ, Sacks FM, Carey VJ, Obarzanek E, Swain JF, et al. (2005) Effects of protein, monounsaturated fat, and carbohydrate intake on blood pressure and serum lipids: results of the OmniHeart randomized trial. JAMA 294: 2455–2464.
13. Elliott P, Stamler J, Dyer AR, Appel L, Dennis B, et al. (2006) Association between protein intake and blood pressure: the INTERMAP Study. Arch Intern Med 166: 79–87.
14. Micha R, Wallace SK, Mozaffarian D (2010) Red and processed meat consumption and risk of incident coronary heart disease, stroke, and diabetes mellitus: a systematic review and meta-analysis. Circulation 121: 2271–2283.
15. Roussell MA, Hill AM, Gaugler TL, West SG, Heuvel JP, et al. (2012) Beef in an Optimal Lean Diet study: effects on lipids, lipoproteins, and apolipoproteins. Am J Clin Nutr 95: 9–16.
16. Roussell MA, Hill AM, Gaugler TL, West SG, Ulbrecht JS, et al. (2014) Effects of a DASH-like diet containing lean beef on vascular health. J Hum Hypertens.
17. Whiteman D, Muir J, Jones L, Murphy M, Key T (1999) Dietary questions as determinants of mortality: the OXCHECK experience. Public Health Nutr 2: 477–487.
18. (1989) The Atherosclerosis Risk in Communities (ARIC) Study: design and objectives. The ARIC investigators. Am J Epidemiol 129: 687–702.
19. Willett WC, Sampson L, Stampfer MJ, Rosner B, Bain C, et al. (1985) Reproducibility and validity of a semiquantitative food frequency questionnaire. Am J Epidemiol 122: 51–65.
20. Willett W, Stampfer MJ (1986) Total energy intake: implications for epidemiologic analyses. Am J Epidemiol 124: 17–27.
21. White AD, Folsom AR, Chambless LE, Sharret AR, Yang K, et al. (1996) Community surveillance of coronary heart disease in the Atherosclerosis Risk in Communities (ARIC) Study: methods and initial two years' experience. J Clin Epidemiol 49: 223–233.
22. Richardson MT, Ainsworth BE, Wu HC, Jacobs DR Jr, Leon AS (1995) Ability of the Atherosclerosis Risk in Communities (ARIC)/Baecke Questionnaire to assess leisure-time physical activity. Int J Epidemiol 24: 685–693.
23. Hu FB, Stampfer MJ, Manson JE, Ascherio A, Colditz GA, et al. (1999) Dietary saturated fats and their food sources in relation to the risk of coronary heart disease in women. Am J Clin Nutr 70: 1001–1008.
24. Hu FB, Bronner L, Willett WC, Stampfer MJ, Rexrode KM, et al. (2002) Fish and omega-3 fatty acid intake and risk of coronary heart disease in women. JAMA 287: 1815–1821.
25. Snowdon DA, Phillips RL, Fraser GE (1984) Meat consumption and fatal ischemic heart disease. Prev Med 13: 490–500.
26. Appel LJ, Moore TJ, Obarzanek E, Vollmer WM, Svetkey LP, et al. (1997) A clinical trial of the effects of dietary patterns on blood pressure. DASH Collaborative Research Group. N Engl J Med 336: 1117–1124.
27. Sacks FM, Campos H (2010) Dietary therapy in hypertension. N Engl J Med 362: 2102–2112.
28. Hu FB, Stampfer MJ, Manson JE, Rimm E, Colditz GA, et al. (1997) Dietary fat intake and the risk of coronary heart disease in women. N Engl J Med 337: 1491–1499.
29. Oh K, Hu FB, Manson JE, Stampfer MJ, Willett WC (2005) Dietary fat intake and risk of coronary heart disease in women: 20 years of follow-up of the nurses' health study. Am J Epidemiol 161: 672–679.
30. Vega-Lopez S, Lichtenstein AH (2005) Dietary protein type and cardiovascular disease risk factors. Prev Cardiol 8: 31–40.
31. Lichtenstein AH, Appel LJ, Brands M, Carnethon M, Daniels S, et al. (2006) Diet and lifestyle recommendations revision 2006: a scientific statement from the American Heart Association Nutrition Committee. Circulation 114: 82–96.
32. Chowdhury R, Warnakula S, Kunutsor S, Crowe F, Ward HA, et al. (2014) Association of dietary, circulating, and supplement fatty acids with coronary risk: a systematic review and meta-analysis. Ann Intern Med 160: 398–406.
33. Ramsden CE, Zamora D, Leelarthaepin B, Majchrzak-Hong SF, Faurot KR, et al. (2013) Use of dietary linoleic acid for secondary prevention of coronary heart disease and death: evaluation of recovered data from the Sydney Diet Heart Study and updated meta-analysis. BMJ 346: e8707.
34. Kumanyika SK, Van Horn L, Bowen D, Perri MG, Rolls BJ, et al. (2000) Maintenance of dietary behavior change. Health Psychol 19: 42–56.
35. Luepker RV, Murray DM, Jacobs DR, Jr., Mittelmark MB, Bracht N, et al. (1994) Community education for cardiovascular disease prevention: risk factor changes in the Minnesota Heart Health Program. Am J Public Health 84: 1383–1393.
36. Perk J, De Backer G, Gohlke H, Graham I, Reiner Z, et al. (2012) European Guidelines on cardiovascular disease prevention in clinical practice (version 2012). The Fifth Joint Task Force of the European Society of Cardiology and Other Societies on Cardiovascular Disease Prevention in Clinical Practice (constituted by representatives of nine societies and by invited experts). Eur Heart J 33: 1635–1701.
